# Primary Cutaneous Carcinosarcoma: A Literature Review and a Report of Two Cases

**DOI:** 10.7759/cureus.96225

**Published:** 2025-11-06

**Authors:** Abdelrahman Ibrahim, Weiguang Ho, Narayanan Viswanathan, Anais Rosich-Medina

**Affiliations:** 1 Plastic and Reconstructive Surgery, Norfolk and Norwich University Teaching Hospitals NHS Foundation Trust, Norwich, GBR

**Keywords:** carcinosarcomas, cutaneous carcinosarcoma, : metaplastic breast carcinoma, metastatic skin cancer, sarcomatoid cancer of the skin

## Abstract

Carcinosarcomas are rare malignant tumors with both epithelial and mesenchymal components, classified into visceral and cutaneous subtypes. Primary cutaneous carcinosarcoma (PCS) is exceptionally rare, and robust evidence to guide its management is lacking. This study aims to consolidate current knowledge on PCS through two new case reports and a comprehensive literature review, offering insights into its pathogenesis, clinical features, and management strategies. Two cases of PCS managed by a multidisciplinary team were reported, detailing clinical, histopathological, and immunohistochemical findings. A systematic literature review of PCS cases published between 2001 and 2021 was conducted, adhering to the Preferred Reporting Items for Systematic Reviews and Meta-Analyses (PRISMA) guidelines, and collating data on clinical presentation, histology, treatment, and outcomes. Literature analysis identified 74 patients across 42 case reports. Histopathology reveals a frequent epithelial component of squamous cell carcinoma (43.2%) or basal cell carcinoma (27.0%). Immunohistochemistry (IHC) aids diagnosis, with vimentin marking mesenchymal components. Surgical excision is the primary treatment; recurrence occurs in 16.2% of cases. Prognosis varies, with better outcomes linked to epithelial subtypes. PCS remains a diagnostic and therapeutic challenge due to its rarity and lack of standardized guidelines. This study highlights the importance of histopathology, IHC, and multidisciplinary management.

## Introduction

Carcinosarcomas are rare malignant tumors characterized by biphasic components of carcinoma and sarcoma. These tumors are classified into visceral and cutaneous subtypes, each exhibiting distinct demographic and prognostic features [1]. Cutaneous carcinosarcomas (CCS) were first described in 1972 by Dawson [2]. However, the terminology surrounding this neoplasm is highly inconsistent, with a variety of synonymous terms in the literature, including carcinosarcoma (CS), sarcomatoid carcinoma, spindle cell sarcoma, bipolar carcinoma, malignant mixed tumor, and metaplastic carcinoma. This terminological diversity complicates understanding and hampers comparative analysis of the condition.

Despite their rarity, cutaneous carcinosarcomas are further subclassified based on the specific epithelial components involved, such as squamous cell carcinoma or basal cell carcinoma. This subclassification is critical for understanding the tumor’s behavior and predicting its prognosis [3].

The pathogenesis of carcinosarcomas remains a topic of debate, with three prevailing hypotheses proposed in the literature. The “divergent hypothesis” suggests that the tumor originates from pluripotent stem cells capable of differentiating into both ectodermal and mesenchymal tissues. The “convergence hypothesis” posits that the tumor arises from separate mesenchymal and epithelial progenitor cells that differentiate into a multiclonal neoplasm. Meanwhile, the “collision hypothesis” theorizes that carcinosarcomas result from two synchronous primary tumors of carcinoma and sarcoma origins [4].

Diagnosis of carcinosarcomas is histological, relying on the identification of carcinoma (typically squamous cell carcinoma or basal cell carcinoma) alongside a spindle cell sarcomatous component. Immunohistochemistry (IHC) further aids in confirming the diagnosis, particularly in poorly differentiated tumors. Markers such as p63 for epithelial components and vimentin for mesenchymal components are particularly reliable. A summary of reported IHC findings is included (List 1).

A major challenge in the study and management of carcinosarcomas is the rarity of cases, resulting in a paucity of robust evidence to guide clinical management, follow-up strategies, and diagnosis. This rarity may also contribute to underdiagnosis and underreporting.

To address this gap in knowledge, we present two cases of primary cutaneous carcinosarcoma (PCS) managed within a multidisciplinary skin cancer team. Our aim is to consolidate findings from the literature and propose a comprehensive management plan for this exceptionally rare entity.

## Case presentation

Case 1

**Figure 1:**
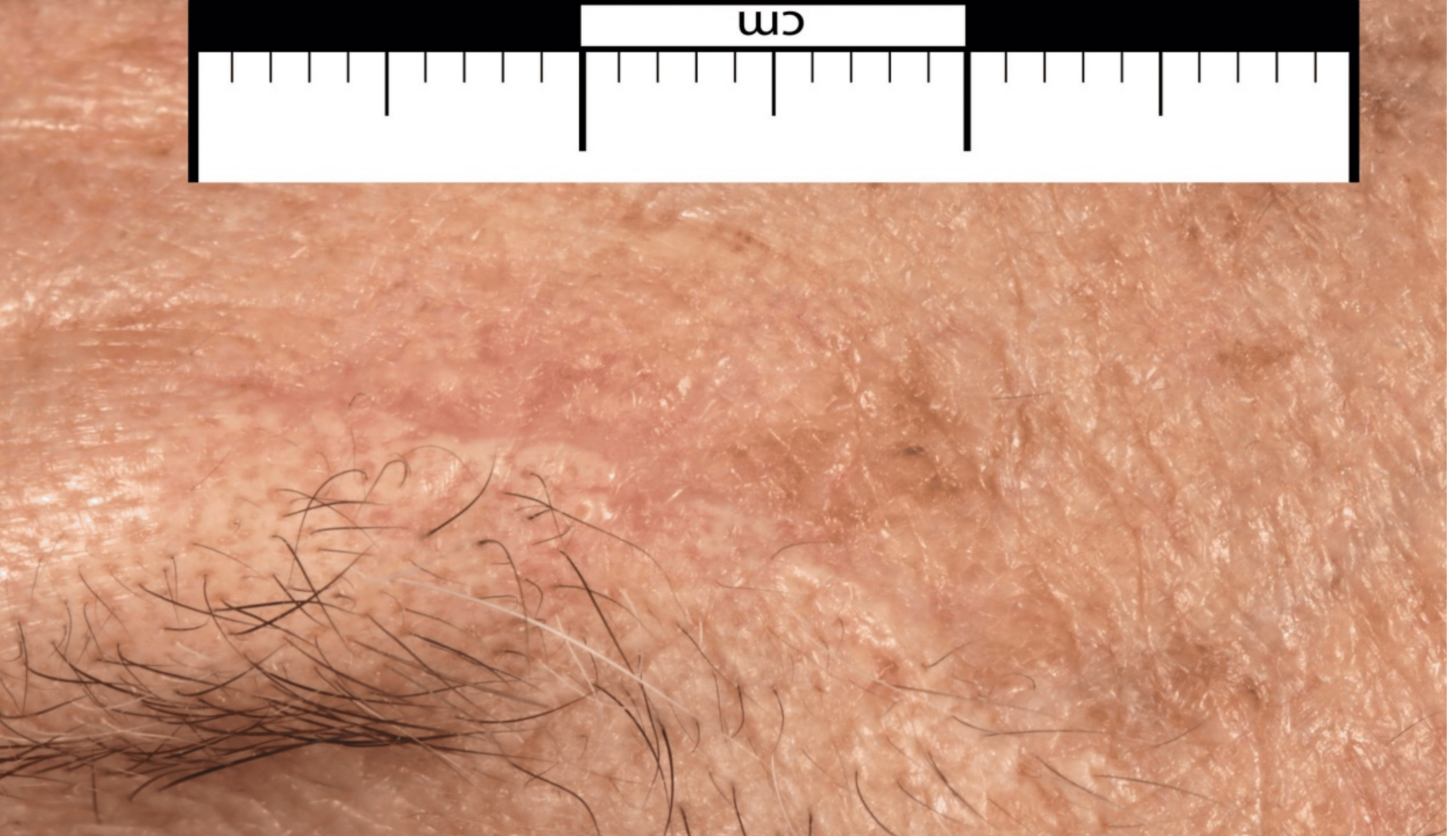
Close-up view of the lesion over the left eyebrow with measured dimensions

A 77-year-old male patient presented with a raised, crusting, bleeding, non-tender lesion measuring 6 x 7 mm on his left eyebrow (Figure 1). There was no cervical lymphadenopathy or organomegaly. No other concerning skin lesions. The lesion had been present for several years with a recent increase in size. The lesion had well-defined borders and exhibited characteristics suggestive of squamous cell carcinoma. The patient’s comorbidities were significant of low body mass index (BMI) of 17, rheumatoid arthritis, myelofibrosis, and Sjögren’s disease.

**Figure 2:**
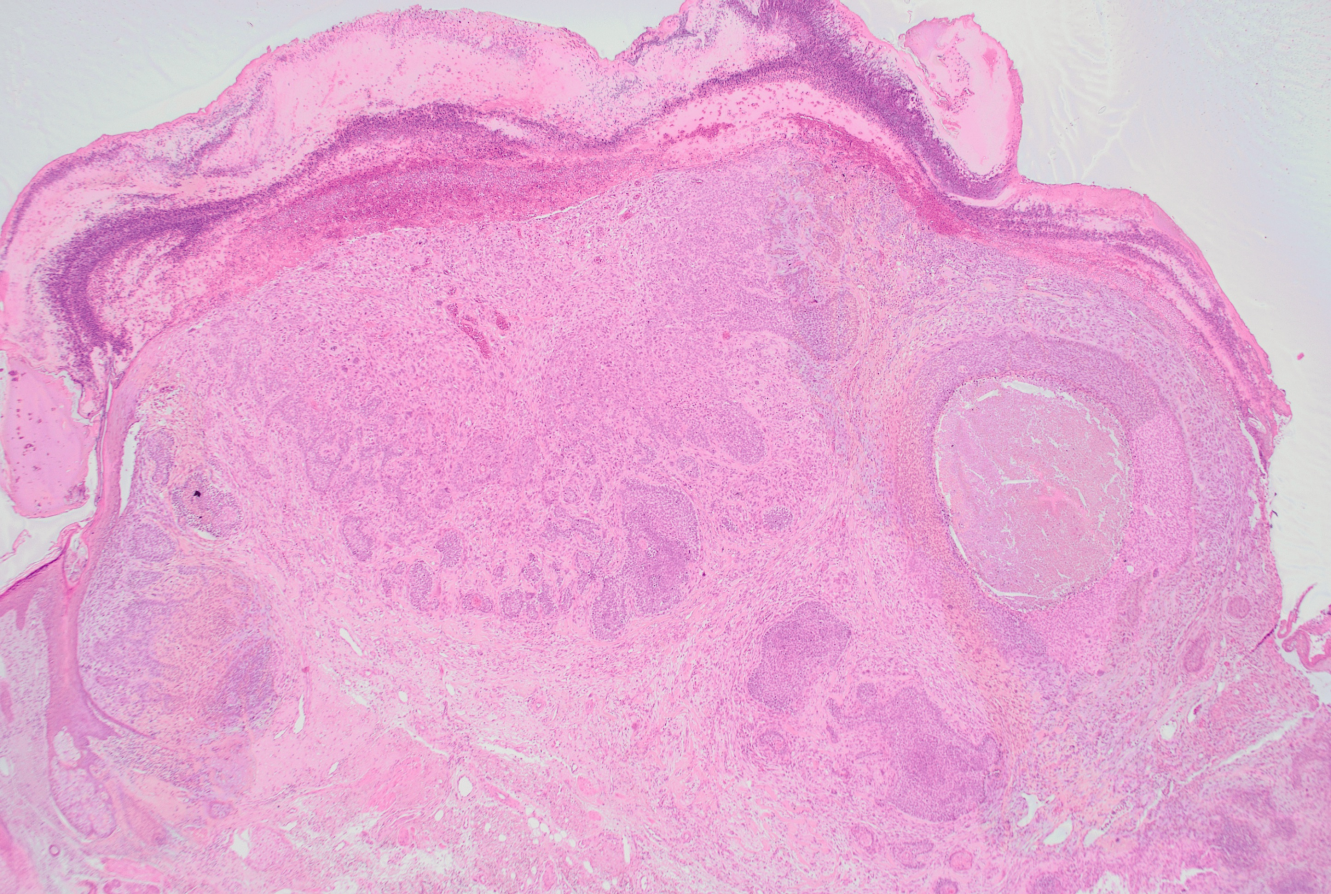
Hematoxylin and eosin staining with no magnification of the first specimen This stained section of cutaneous carcinosarcoma shows biphasic morphology with malignant epithelial and spindle-cell mesenchymal components

**Figure 3:**
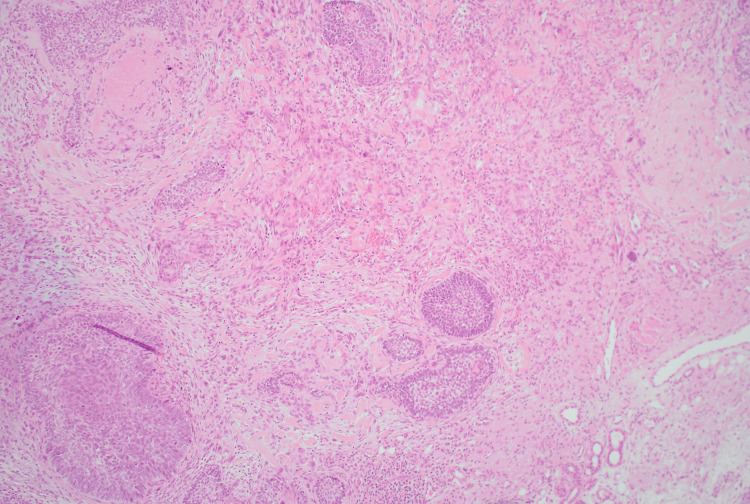
At low-power magnification (H&E, ×4) of the first specimen H&E: hematoxylin and eosin There are malignant epithelial islands, seen embedded within a spindle-cell stroma, consistent with a biphasic tumour

**Figure 4:**
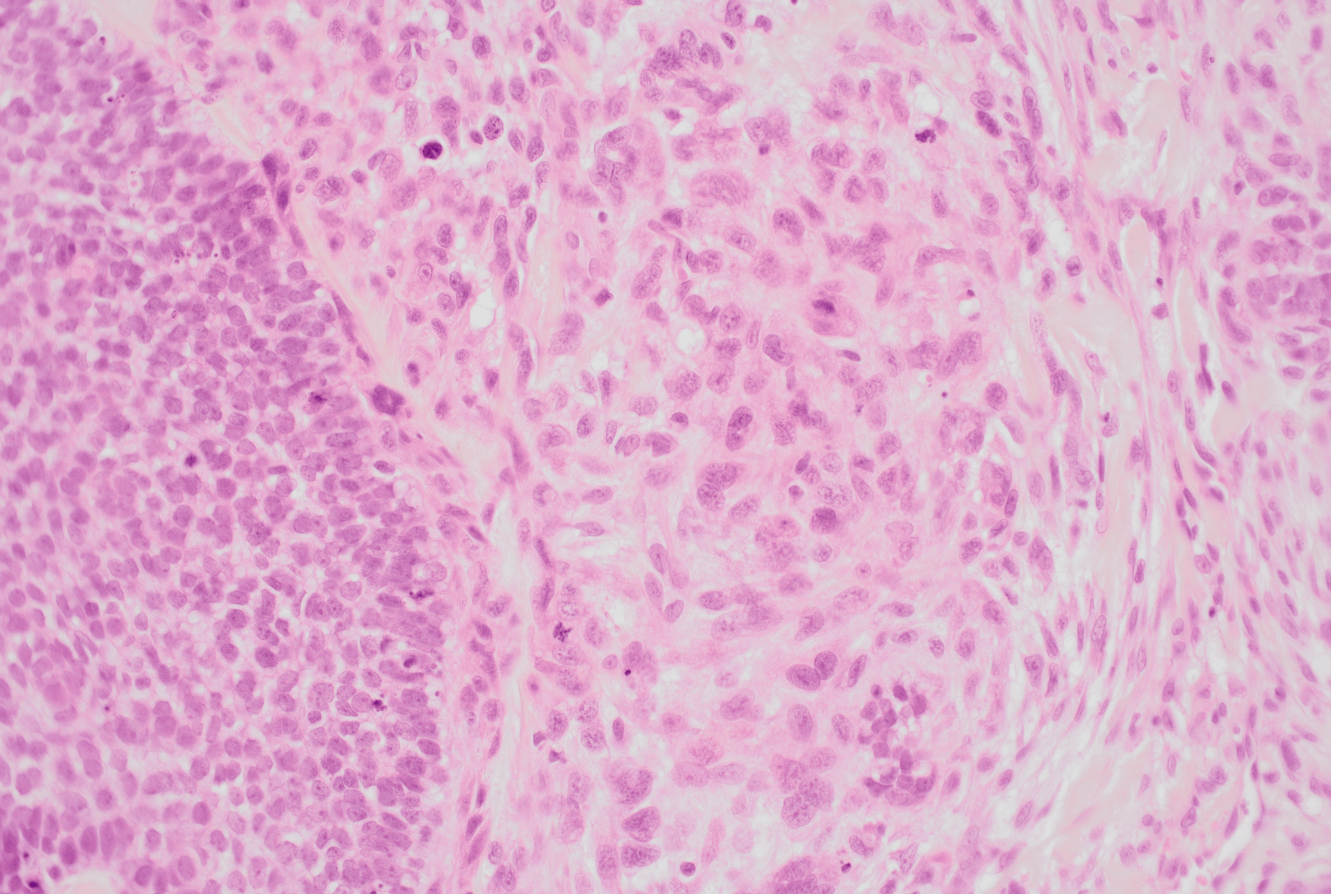
High-power view (H&E, ×40) of the first specimen H&E: hematoxylin and eosin This slide is showing malignant spindle cells with marked nuclear pleomorphism, vesicular chromatin, prominent nucleoli, and frequent atypical mitoses adjacent to epithelial tumour nests

An excisional biopsy was performed, revealing features indicative of both basal cell carcinoma and spindle cell stroma. The biopsy demonstrated a 1 mm deep margin free of tumour, while the nearest peripheral margin measured 2 mm. The tumour is composed of basaloid nests with peripheral palisading (basal cell carcinoma component) and cellular spindled stroma with multinucleate giant cells, bizarre mitotic figures (sarcomatous stroma) (hematoxylin and eosin (H&E) stain) (Figures 2-4). Immunohistochemical staining revealed positive expression of BerEP4 in the basal cell carcinoma component, while the atypical stromal component displayed strong positivity for vimentin. These findings were further supported by an expert opinion obtained from the Dermatopathology Department at St. Thomas' Hospital in London. Additionally, additional stains confirmed positive results for MNF116 in the epithelial components. 

The patient passed away after a three-month follow-up period, and no evidence of tumour recurrence or metastasis was detected during the coroner's examination.

Case 2

**Figure 5:**
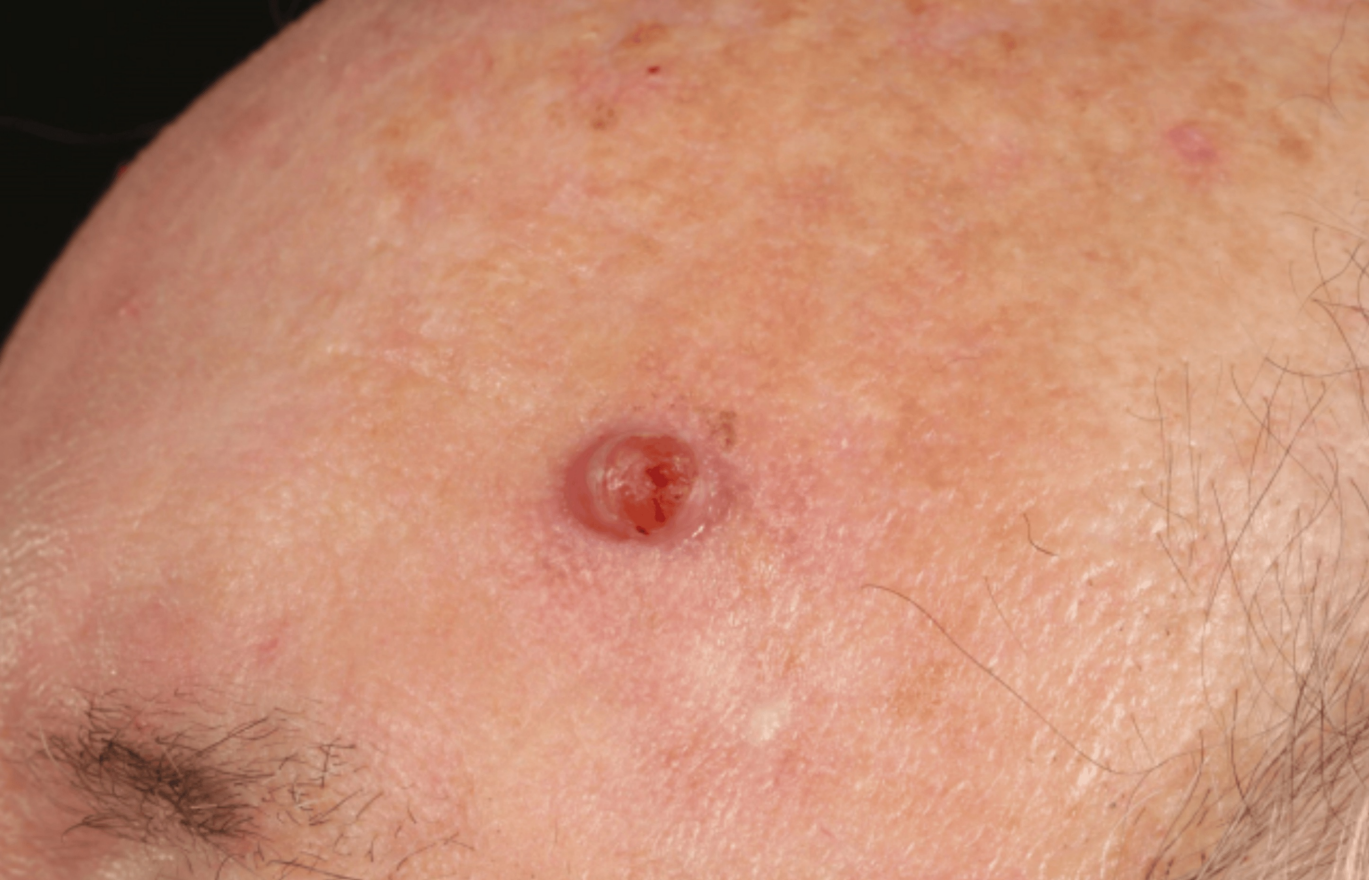
Left forehead lesion, close-up view demonstrating location

**Figure 6:**
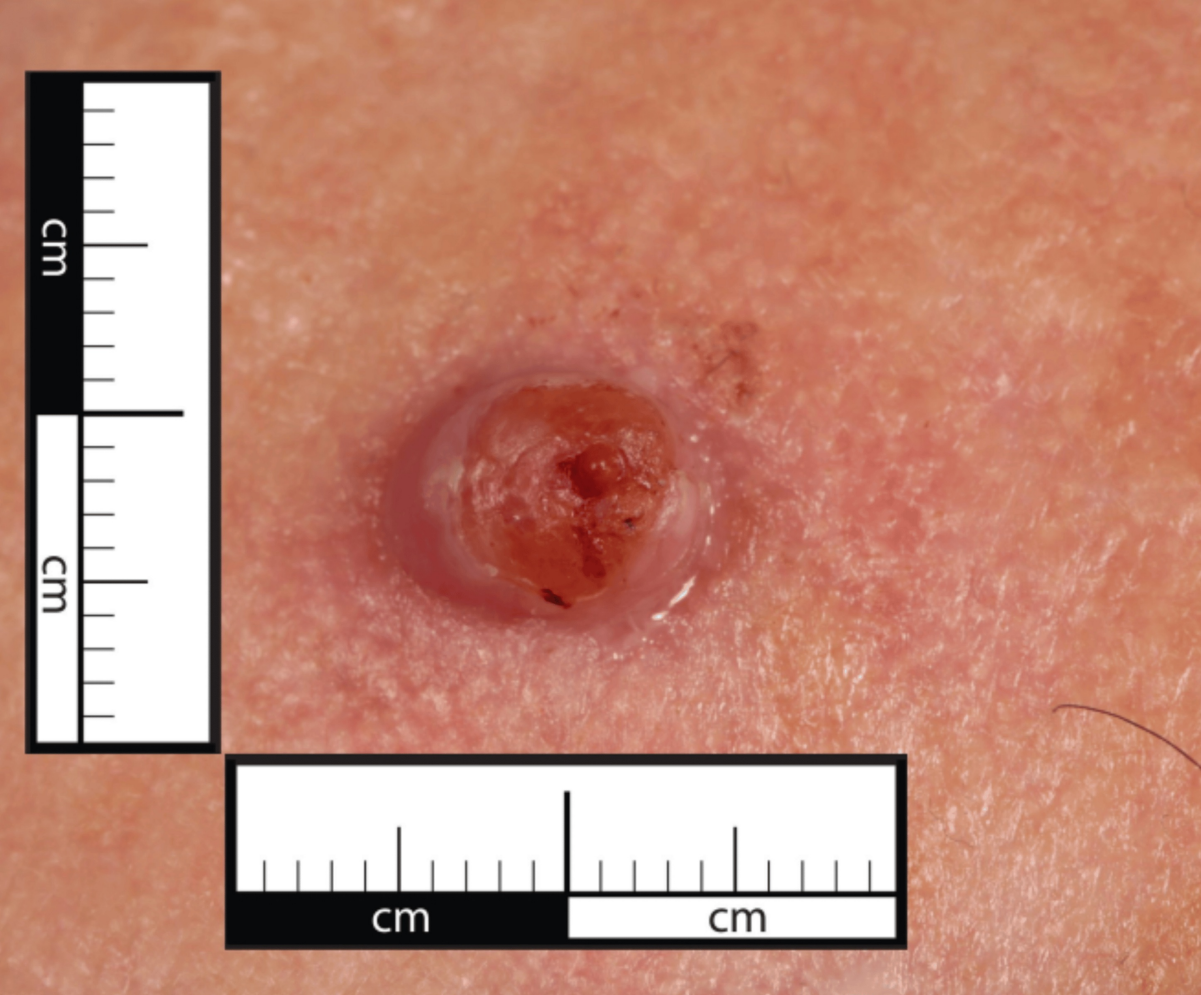
Close-up view of the lesion on the left forehead showing its size and surface characteristics

An 82-year-old male patient presented with a three-month history of a rapidly growing, mobile, raised ulcerated 8 x 7 mm lesion with defined borders in the left temple with no other skin lesions (Figures 5, 6). There were no palpable cervical lymph nodes and no organomegaly. Further assessment of dermoscopy showed features suggestive of a squamous cell carcinoma. An excisional biopsy was performed, and the defect resurfaced with a full-thickness skin graft.

**Figure 7:**
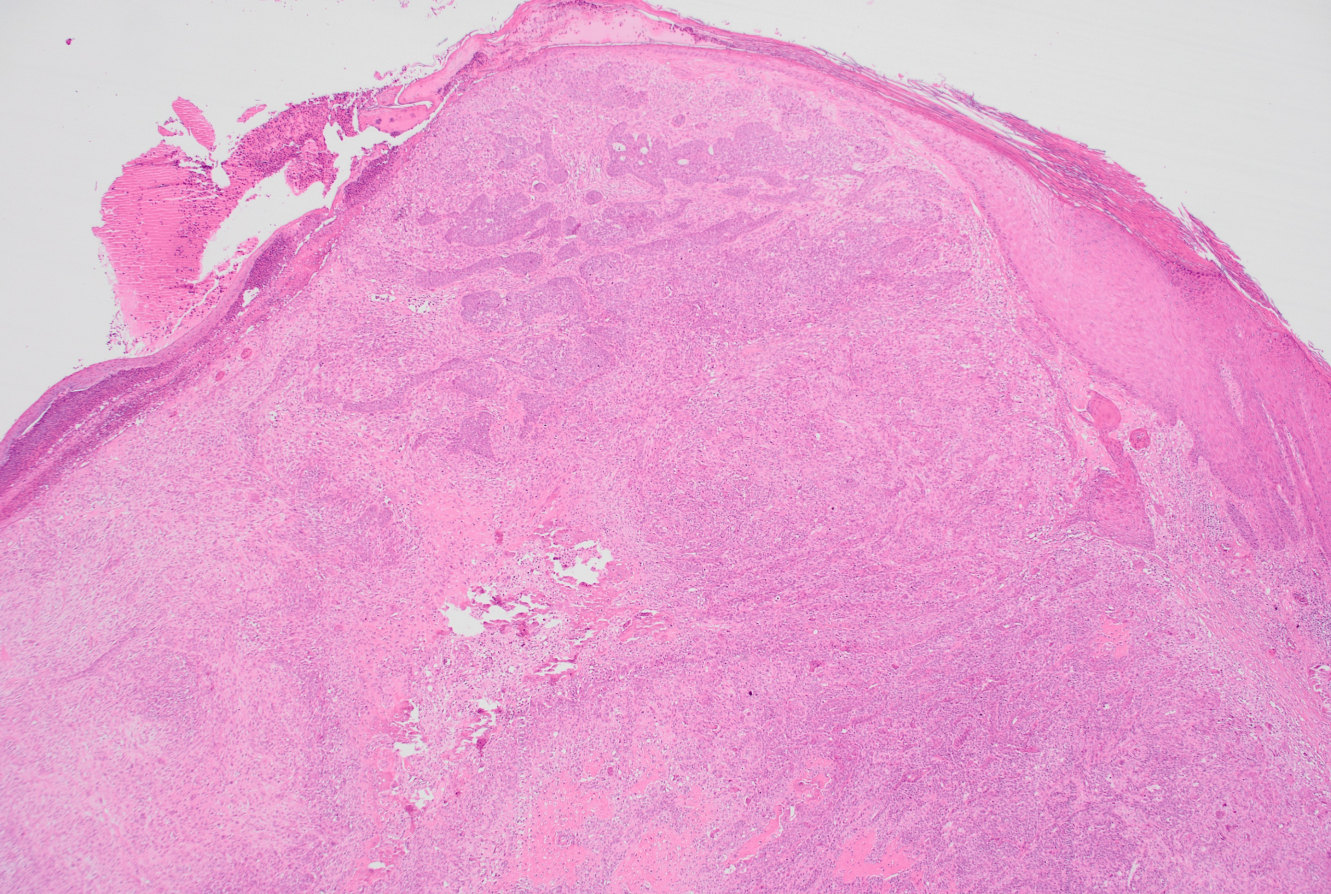
Hematoxylin and eosin with no magnification of the second specimen H&E: hematoxylin and eosin Low-power view (H&E, ×1) showing a biphasic neoplasm composed of epithelial nests and spindle-cell stroma

**Figure 8:**
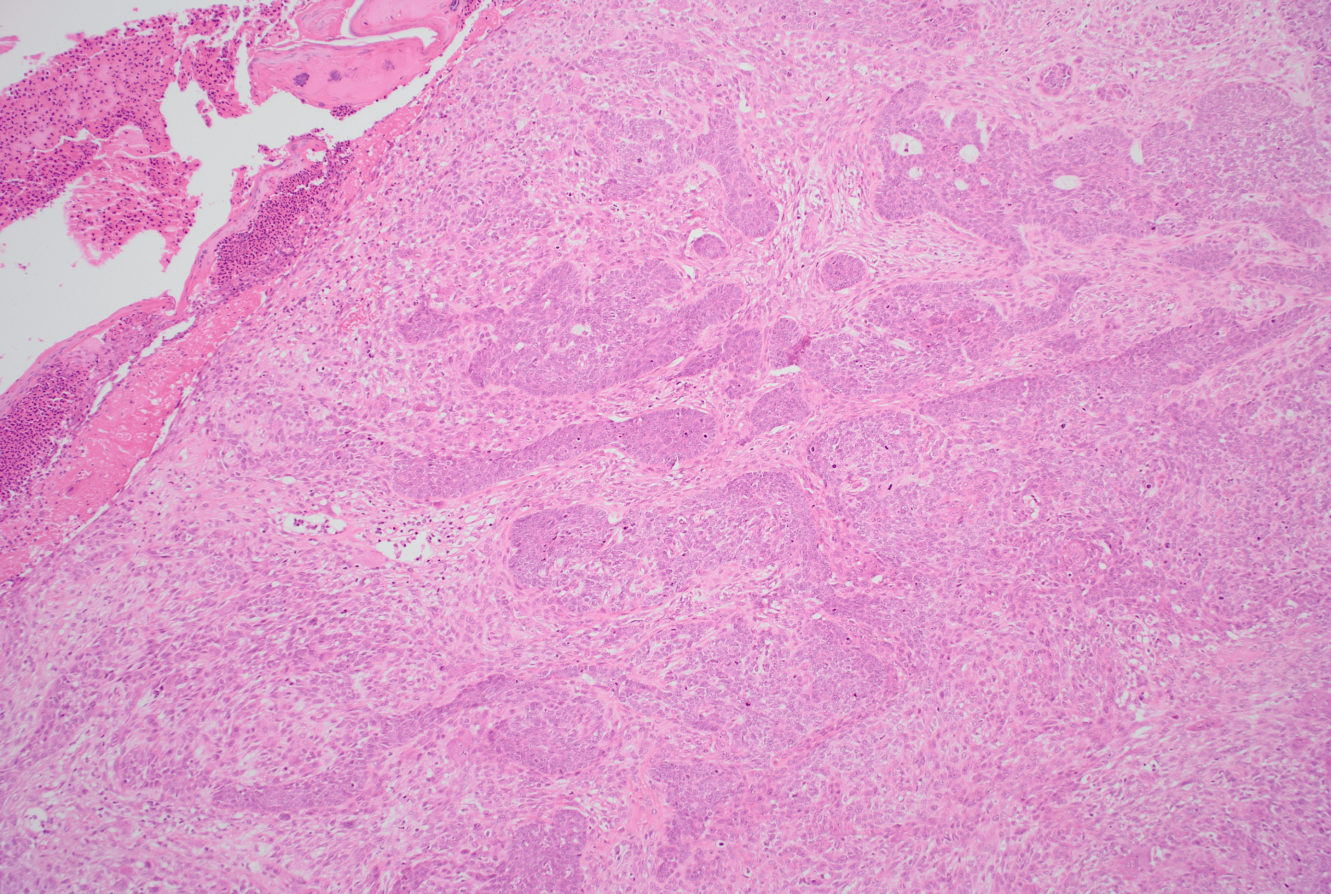
At low-power magnification (H&E, ×4), of the first specimen H&E: hematoxylin and eosin The tumour from the second patient demonstrates a biphasic neoplasm composed of both epithelial and mesenchymal components. At four times magnification, basaloid epithelial nests with peripheral palisading, consistent with a basal cell carcinoma component, are evident within a cellular spindle-cell stroma. The sarcomatous stroma shows nuclear pleomorphism, scattered atypical mitotic figures, and multinucleated giant cells

**Figure 9:**
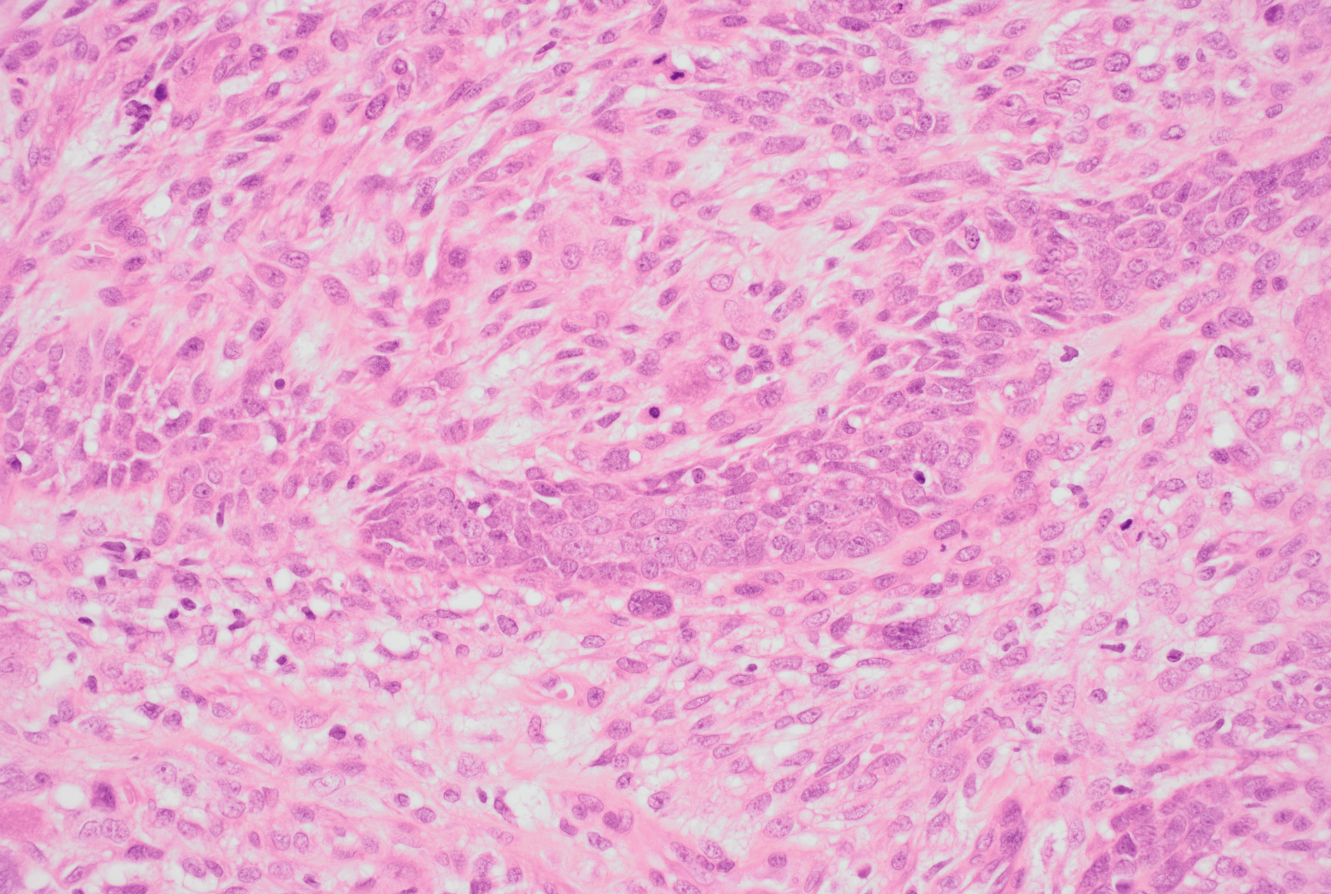
Hematoxylin and eosin staining at higher magnification (10X) of the second specimen H&E: hematoxylin and eosin This demonstrates epithelial nests consistent with basal cell carcinoma, interspersed with sarcomatous stroma containing pleomorphic spindle cells, multinucleated giant cells, and atypical mitotic figures

The histopathology examination revealed sheets and islands of basaloid cells merging with spindle cell stroma with evidence of malignancy, pleomorphism, increased mitotic figures, and an infiltrative growth pattern. The tumour shows a basaloid component with anastomosing islands of sheets and islands (basal cell carcinoma component) merging with cellular stroma composed of spindles to epithelioid cells with areas of osteosarcomatous differentiation (sarcomatous stroma) (H&E stain) (Figures 7-9). Notably, there was evidence of calcification and ossification noted within the centre of the islands, together with palisading osteoclast-like multinucleate giant cells. The lesion measures a thickness of 6.5 mm with a clearance of 12 mm in the peripheral margin and 1 mm to the deep margin. Immunohistochemical staining revealed positive expression of BerEP4 in the basaloid cells, and vimentin highlights in the spindle cell component. Further staging imaging, including CT neck, chest, abdomen, and pelvis, and MRI head scans, was unremarkable. The skin multidisciplinary meeting decision recommended that the patient have radiotherapy for a close deep margin. This patient completed a one-year follow-up with no evidence of recurrence.

## Discussion

Literature review

Methods

A literature search was performed in PubMed with the keyword “carcinosarcoma” for the period between 2001 and 2021. All cohort studies, retrospective reviews, case series, and case reports on PCS were accepted for review. Exclusion of publications was in accordance with Preferred Reporting Items for Systematic Reviews and Meta-Analyses (PRISMA) guidelines. Clinical details, histopathological features, management, and prognosis of each patient in the included publications were collated into a table along with the reported cases from this case report for comparison.

Results

We collected a total of 42 case reports involving 74 patients (Table 1). The prevalence of this condition appears to be higher among males compared to females, with a male-to-female ratio of 2:1 in head and neck cases (67.7%) and 9.5% in the trunk. Notably, this ratio increases to 3:1 in cases involving the extremities, accounting for 23% of the occurrences. The lesions predominantly manifest as substantial growths, exhibiting an average dimension of 35.7 mm. These dimensions, however, vary across different anatomical regions.

**Table 1:** Summary of the clinical and pathological findings of reported cases of cutaneous carcinosarcoma NR: not reported; N/A: not applicable; SCC: squamous cell carcinoma; BCC: basal cell carcinoma; SCAP: syringocystadenocarcinoma papilliferum

Case	Reference	Case no	Age	Gender	Anatomical region	Metastasis	Follow up period	Epithelial component	Mesenchymal component
1	García-Souto [5]	1	91	Male	Forehead	N/A	Lost to follow up	SCC	High-grade epithelioid sarcoma
		2	89	Male	Leg	No	31	BCC	Mixed sarcoma (fusocellular and myxoid)
		3	80	Male	Scalp	Yes	37	SCC	High-grade fusocellular sarcoma
		4	55	Male	Nose	No	78	Basosquamous	Atypical fibroxanthoma-like sarcoma
		5	87	Female	Trunk	No	17	BCC	Fusocellular sarcoma with osteoid differentiation
		6	85	Female	Cheek	No	11	SCC	Mixed sarcoma (fusocellular and myxoid)
		7	44	Male	Scalp	No	31	SCC	Atypical fibroxanthoma-like sarcoma
		8	46	Male	Leg	No	61	Mucoepidermoid	Rhabdomyosarcoma
2	Kim [6]	1	34	Female	Nose	NR	12	Trichoblastic carcinoma	NR
3	Giang [7]	1	80	Male	Ear	NR	NR	BCC	NR
4	Underwood [8]	1	79	Male	Scalp	NR	12	BCC	NR
5	Luong [9]	1	74	Female	Scalp	NR	45	Pilomatrical carcinoma	Undifferentiated pleomorphic sarcoma
6	Davis [10]	1	62	Male	Leg	N/A	3	BCC	Osteosarcoma
7	Gates [11]	1	9	Female	Neck	N/A	NR	Pilomatrical carcinoma	NR
8	Song [12]	1	57	Male	Scalp vertex	No	12	SCC	Unknown
		2	75	Female	Scalp	No	NR	BCC	Unknown
9	Kwon [13]	1	69	Male	Forearm	No	6	Porocarcinoma	NR
10	Mijušković [14]	1	58	Female	Back	Yes - death	8	Basosquamous	Undifferentiated pleomorphic sarcoma
11	Hormuzdi [15]	1	14	Male	Neck	N/A	NR	NR	NR
12	Gamret [16]	1	66	Male	Hand	Yes - Lung	NR	SCC	Undifferentiated-appearing spindle cells
13	West [17]	1	82	Male	Medial canthus	No	7	SCC	Malignant spindle cell proliferation
14	Alegría-Landa [18]	1	90	Female	Scalp	N/A	12	Syringocystadenocarcinoma papilliferum	NR
15	Kwak [19]	1	77	Male	Penile tip	N/A	NR	SCC	Malignant dysplastic spindle cells
		2	96	Female	Scalp	N/A	NR	SCC	Malignant dysplastic spindle cells
		3	87	Female	Cheek	N/A	NR	SCC	Malignant dysplastic spindle cells
		4	43	Male	Cheek	N/A	NR	SCC	Malignant dysplastic spindle cells
		5	74	Female	Neck	N/A	NR	SCC	Malignant dysplastic spindle cells
		6	96	Male	Auricle	N/A	NR	SCC	Malignant dysplastic spindle cells
		7	68	Female	Calf	N/A	NR	SCC	Malignant dysplastic spindle cells
		8	45	Male	Cheek	N/A	NR	SCC	Malignant dysplastic spindle cells
		9	63	Male	Thigh	N/A	NR	SCC	Malignant dysplastic spindle cells
		10	79	Male	Cheek	N/A	NR	BCC	Malignant dysplastic spindle cells
		11	58	Male	Scalp	N/A	NR	SCC	Malignant dysplastic spindle cells
16	Leecy [20]	1	87	Female	Hand	N/A	6	Pilomatrical carcinoma	Undifferentiated pleomorphic sarcoma
17	Ruiz-Villaverde [21]	1	85	Female	Forehead	N/A	NR	BCC	Spindle cells with moderate pleomorphism
18	Okhremchuk [22]	1	83	Male	Back	N/A	12	Trichoblastic carcinoma (separate BCC)	NR
19	Pazzini [23]	1	56	Female	Neck	N/A	36	BCC, SCC, sebaceous carcinoma	Osteosarcoma
20	Lim [24]	1	54	Male	Handx2	N/A	NR	SCC	Atypical spindle cell proliferation
21	Suzuki [25]	1	94	Male	Cheek	N/A	24	BCC	NR
22	Wollina [26]	1	80	Female	Leg	N/A	NR	SCC	Undifferentiated sarcoma
23	Fernandez-Flores [27]	1	78	Male	Hand	N/A	8	Pilomatrix carcinoma	NR
24	Clark [28]	1	77	Male	Cheek	N/A	2	Pilomatrical/BCC	Undifferentiated spindle cell sarcoma
		2	82	Male	Scalp	NR	18	SCC	Undifferentiated spindle cell sarcoma
		3	81	Male	Forehead	N/A	NR	Trichoblastic	Undifferentiated spindle cell sarcoma
		4	85	Male	Finger	N/A	NR	Trichoblastic	Undifferentiated spindle cell sarcoma
		5	73	Male	Ear	N/A	8	BCC	Undifferentiated spindle cell sarcoma
		6	90	Male	ear	N/A	10	BCC	Undifferentiated spindle cell sarcoma
25	Suyama [29]	1	73	Male	Left preauricular	N/A	8	Pilomatrical carcinoma	NR
26	Colston [30]	1	65	Male	Forehead	N/A	4	Trichoblastic carcinoma	Malignant spindle cell proliferation
27	Xu [31]	1	76	Male	Scalp	N/A	12	BCC	Osteosarcoma
28	Zbacnik [32]	1	71	Male	Arm	N/A	8	BCC	Spindle cell sarcoma
29	Bourgeault [33]	1	91	Female	Nose	N/A	12	BCC	Fibrosarcomatous stroma
30	Harvey [34]	1	87	Male	Ear	N/A	12	BCC	Undifferentiated spindle cell malignancy
		2	74	Male	Ear	N/A	2	BCC	undifferentiated malignant spindle cell stroma
		3	80	Male	Ear	N/A	27	BCC	Pleomorphic spindle cells, scattered multinucleate tumor giant cells
		4	73	Male	Calf	N/A	Lost to follow up	BCC	Spindled cells on variably fibrous to myxoid backgroind
31	Fujii [35]	1	70	Male	Arm	N/A	12	SCC	Sarcomatous spindle-cell stromal component
32	Kiuru [36]	1	72	Male	Forearm	Yes. , Death	2	BCC	Undifferentiated spindle cells
33	Müller [37]	1	86	5 Males and 1 Female	Forehead	N/A	7	SCC	Myogenic sarcoma
		2	76		Back		21	SCC	Neurogenic sarcoma
		3	53		Ear	N/A	31	SCC	Rhabdomyosarcoma
		4	84		Eye brow	N/A	13	SCC	Malignant fibrous histiocytoma-like, myxoid-pleomorphic type, neurogenic sarcoma
		5	86		Forehead	N/A	8	SCC	Myogenic sarcoma
		6	74		Temple	N/A	0	SCC	Myofibroblastic sarcoma
34	Loh [38]	1	75	Male	Cheek	Yes. Right parotid	12	SCC	NR
35	Hong [39]	1	84	Male	Chest/ Shoulder	N/A	6	SCC	Undifferentiated spindle cells
36	Kwan [40]	1	63	Male	Scalp	N/A	33	SCC	Atypical fibroxanthoma
37	Tse [41]	1	92	Male	Chest	N/A	14	BCC	Osteosarcoma
		2	81	Male	Helix	N/A	6	BCC	Osteosarcoma
38	Sangjin [42]	1	85	Female	Face	N/A	6	SCC	Pleomorphic large spindle cells
39	Chittari [43]	1	87	Female	Hand	Cerebral mets, death after 6 months	6	SCC	Atypical multinucleate giant cells and atypical spindle cells in stroma
40	Lau [44]	1	80	Male	Scalp	Liver mets	6	Merkel cell carcinoma only initially, Merkel cell carcinosarcoma on recurrence	Spindle cell sarcoma
41	Bakhshi [45]	1	45	Female	Scalp	N/A	12	SCAP	Spindle cell differentiation
42	Chou [46]	1	67	Male	Forehead	N/A	12	Sebaceous carcinoma	Spindle cells with hyperchromatic nuclei and numerous mitotic figures

Diagnosis primarily relies on surgical biopsy, with excision biopsy accounting for 17 cases, shave biopsy for six cases, and incisional biopsy for eight cases. IHC played a significant role in diagnosing the condition, as it was utilised in 94.5% of cases. The specifics of the utilised IHC markers are detailed in Table 2. Among the mesenchymal components, vimentin was frequently detected, while beta-catenin appeared in both epithelial and mesenchymal components of carcinosarcomas in four cases. Conversely, desmin and S100 were consistently absent in both the epithelial and mesenchymal components. Expression of SMA exhibited variability across epithelial and mesenchymal components.

**Table 2:** Reported immune histochemistry Summary of reported immunohistochemical markers, categorized into carcinoma-specific and sarcoma-specific markers

Category	Markers
Carcinoma	AE1/AE3, p16, p40, p53, p63, EMA, BCL2, MNF116, cytokeratin (CK), BerEP4, TTF1, CDX2, PSA, S100, KP1, MART, podoplanin, E-cadherin, CEA, CK5/6, CK7, CK14, CK19, CK903, CK20, CD10, mammaglobin, alpha-1 antitrypsin, CGDFP-15, CA 19.9, vimentin
Sarcoma	SMA, myosin, desmin, actin, myogenin, S100, CD34, CD117 (c-kit), CD99, HMB45, SOX-10, vimentin
Others	OSCAR, neuron-specific enolase, beta-catenin, leucocyte common antigen (LCA), factor VIII, factor XIIIa, synaptophysin, chromogranin, transcription termination factor 1, melan A, tyrosinase

The epithelial aspect of the carcinosarcoma often comprised squamous cell carcinoma (43.2%) and basal cell carcinoma (27.0%). Notably, cases of epithelial carcinosarcomas outnumbered adnexal carcinosarcomas, constituting 71.2% versus 28.8%, respectively.

Staging investigations were performed in eight out of the 74 cases, with four involving computed tomography scans. The primary treatment approach revolved around surgical excision, with reconstruction undertaken when necessary. Recurrence was documented in 12 cases, accounting for 16.2% of instances.

Definition

The National Cancer Institute at the National Institutes of Health defines carcinosarcoma as a malignant tumour that is a mixture of cancer of epithelial tissue (carcinoma) and cancer of connective tissue (sarcoma) [47]. It is also referred to as metaplastic carcinoma, sarcomatous (or sarcomatoid) carcinoma, pseudosarcoma, or biphasic sarcomatoid carcinoma in the published literature.

Pathogenesis

The exact aetiology of PCS is not established; however, three theories exist for tumourigenesis, including collision, conversion, and divergence theories. The collision theory, also referred to as the epithelial-mesenchymal transition (EMT) phenomenon, proposes that carcinosarcoma may arise from a metaplastic transformation of epithelial carcinoma. Although extensive mechanisms and various molecular pathways underlying the collision theory have been delineated through in vitro experiments, and prominent regulatory genes involved have been identified (such as Twist, Snail, and Slug), the validity of the cancer-associated collision theory remains a subject of debate. Some pathologists remain sceptical regarding the relevance of the EMT phenomenon in the context of cancer [48,49]. This notion aligns with the findings of Sung et al., where carcinosarcomas consistently demonstrated diminished or absent expression of epithelial markers (E-cadherin, claudin-3, and claudin-4), along with varying levels of expression of mesenchymal markers (S100A4, α-SMA, PDGFRα, and β-catenin). In contrast, the expression of the mesenchymal marker vimentin appeared to exhibit greater consistency. However, the underlying reasons for this heterogeneous expression pattern remain unclear. EMT-associated transcription factors, including Snail1, SIP1, ZEB1, Twist1, and Slug, have been detected in carcinosarcomas, yet their expression patterns lack consistency, even among samples originating from the same organ [50].

The divergence theory proposes that carcinosarcoma originates from a monoclonal source. This concept of differentiation from a solitary totipotent stem cell is exemplified by a case detailed by Paniz-Mondolfi et al., where transitional chimeric cells positioned at the epithelial-mesenchymal boundary exhibited ultrastructural features revealing simultaneous epithelial and mesenchymal attributes [51]. Moreover, carcinosarcomas have been identified in other organs with cells originating from an epithelial lineage, such as the oesophagus, gastrointestinal tract, and vulva. However, the current body of evidence remains insufficient to draw definitive conclusions about the aetiological origins and associated risk factors of primary carcinosarcoma.

Diagnosis

Diagnosing primary carcinosarcoma poses challenges due to varying clinical findings in the literature, potentially stemming from diverse aetiological theories. These cutaneous carcinomas may manifest as exophytic growths with surface ulceration, which may or may not bleed upon palpation. Alternatively, they can appear as longstanding lesions that rapidly transform just before presentation [52]. The definitive diagnosis of carcinosarcoma relies on histopathological analysis and molecular biomarker staining. Typically, the epithelial component consists of basal cell carcinoma, followed by various adnexal tumours and squamous cell carcinoma. The mesenchymal component primarily involves undifferentiated sarcoma, followed by atypical fibroxanthoma [3]. Malignant features such as mitotic figures, necrosis, pleomorphic cells, and nuclear atypia should be evident. IHC, utilising stains such as p63 and AE1/AE3, aids in confirming epithelial cell origin. Furthermore, staining with vimentin, CD10, or caldesmon helps distinguish the mesenchymal component of primary carcinosarcoma [53].

Tran et al. proposed a classification attempt for PCS, distinguishing between epithelial and adnexal subtypes [1]. This classification is based on their respective underlying epithelial malignancy subtypes. Epithelial primary carcinosarcoma encompasses basal and squamous cell carcinoma, often arising on sun-damaged skin among elderly men. On the other hand, the adnexal subgroup comprises spiradenocarcinoma, porocarcinoma, proliferating trichilemmal cystic carcinoma, and matrical carcinoma. This adnexal subtype is more commonly observed in younger women (with a mean age of 58 years), characterised by longstanding nodules displaying recent growth [1].

Management

Surgical excision remains the primary treatment approach, with various methods and excision margins reported in this review. While the majority of cases did not require soft tissue reconstruction, there were nine instances (12.1%) where skin grafts or flaps were utilised for reconstructing defects, though the therapeutic benefits remain uncertain. Presently, no consensus exists regarding optimal excision margins and whether wider margins confer improved prognosis. Cervoni et al. documented a case where scalp PCS was effectively treated with radiotherapy alone [53].

We recommend surgical excision, when applicable, with at least 1 cm clinical margins. Where feasible, a 2-3 cm margin should be considered. Postoperative radiotherapy should be considered in cases where the margins are close and subsequent repeat surgery is not possible. Staging CT scans should be offered for all cases. This will serve two purposes: detecting subclinical metastasis and providing insights into disease behaviour in cases of local or distant recurrence.

The prognosis of PCS appears to fluctuate based on its subtype. A meta-analysis by Brasanac et al. revealed recurrence or metastasis rates of 13.6% for PCS tumours with a basal cell carcinoma (BCC) component, 45.4% for tumours exhibiting squamous cell carcinoma (SCC) features, and 52.6% for tumours featuring an adnexal component. Additionally, 14.3% of patients in the SCC group succumbed to the disease, compared to 43.7% in the adnexal group [3].

According to a meta-analysis conducted by Tran et al., epithelial primary carcinosarcoma displays a 70% five-year disease-free survival rate, in contrast to adnexal primary carcinosarcoma, which exhibits a 25% five-year disease-free survival rate [1]. We recommend six-monthly follow-up intervals for five years to assess for recurrence or evidence of distant metastatic spread. However, the long-term prognosis for PCS remains uncertain, warranting further investigation [53].

## Conclusions

Cutaneous carcinosarcomas are a rare entity with potential underdiagnosis and underreporting. They are twice as common in males, with 67.7% of lesions located in the head and neck region. They present as large tumours with an average diameter of 35.7 mm. Diagnosis is histological and relies on IHC. The epithelial components are mainly squamous or basal cell carcinomas in 70.2% of cases. These are aggressive tumours, and surgical excision should be offered as the first line of treatment. The recurrence rate has been reported in 16.2% of cases. Postoperative radiotherapy should be considered in cases where the margins are close and subsequent repeat surgery is not possible. Staging CT scans should be offered for all cases. This will serve two purposes: detecting subclinical metastasis and providing insights into disease behaviour in cases of local or distant recurrence. We recommend six-monthly follow-up intervals for five years to assess for recurrence or evidence of distant metastatic spread. However, the long-term prognosis for PCS remains uncertain, warranting further investigation.
